# Effect of the capsular tension ring on refractive outcome after phacoemulsification

**DOI:** 10.22336/rjo.2021.11

**Published:** 2021

**Authors:** Saadet Gültekin Irgat, Fatih Özcura

**Affiliations:** * Department of Ophthalmology, Kutahya Health Sciences University School of Medicine, Kutahya, Turkey

**Keywords:** capsular tension ring, refraction, phacoemulsification surgery

## Abstract

**Purpose:** To evaluate how capsular tension rings (CTR) affect refractive outcomes following cataract extraction by phacoemulsification complicated by zonular instability.

**Study design:** Retrospective.

**Methods:** In a retrospective case-control study, the 29 eyes of 29 patients with CTRs were compared with the 29 eyes of patients without zonular instability following routine phacoemulsification, with mean arithmetic refractive prediction error (ArRPE) and mean absolute refractive prediction error (AbRPE) as primary outcome measures.

**Results:** A comparison of eyes with and without CTRs according to the SRK/ T formula revealed no statistically significant difference in ArRPE (0.52 vs. 0.45 D, p = 0.570) or AbRPE (0.52 vs. 0.55 D, p = 0.799). Postoperative hypermetropic shift occurred in most cases in both groups, although the mean difference between eyes with CTR (22/ 29) and without CTR (25/ 29) was not statistically significant (p = 0.315).

**Conclusion:** Implanting CTRs did not consistently affect refractive outcomes versus routine phacoemulsification. Results suggest that intraocular lens power can be calculated as usual when CTRs are used.

**Abbreviations:** CTR = capsular tension ring, ArRPE = arithmetic refractive prediction errors, AbRPE = absolute refractive prediction errors, IOL = intraocular lens, WTW = white-to-white, ACD = anterior chamber depths, AL = axial lengths, RPE = refractive prediction error

## Introduction

A well-established tool in the armamentarium of cataract surgeons, capsular tension rings (CTR), allow surgeons to approach zonular weakness with improved safety during complex cataract surgery. Since Hara et al. first proposed introducing a ring into the zonular weak capsular sac in 1991 [**1**], ring designs of various sizes have been developed for CTRs.

CTR is implanted primarily during cataract surgery to stabilize the position of the capsular sac, facilitate phacoemulsification, and keep the intraocular lens (IOL) stable in the postoperative centralized state.

Any cause of regional weakness or loss may be an indication for the placement of CTR. The most common causes of zonular insufficiency are pseudoexfoliation syndrome, trauma, previous ocular surgery (after vitrectomy), hypermature cataract and increased axial length [**2**-**6**]. Less common causes include Marfan’s syndrome, homocystinuria, Weill-Marchesani syndrome, microspherophakia, retinitis pigmentosa, and intraocular neoplasms [**7**-**14**].

The aim of our study was to determine whether implanting CTRs in eyes following cataract surgery affects refractive outcomes and the calculation of intraocular lens (IOL) power.

## Materials and methods

Patients

Patients who underwent phacoemulsification in our clinic between January 2016 and January 2019 were divided into two groups - 29 who received CTRs due to zonular instability following phacoemulsification (i.e., Group 1) and 29 without zonular instability and thus without CTRs (i.e., Group 2) - for a total sample of 58 patients. Group 1 was matched in age and sex with Group 2, the control group, and all the patients in both groups underwent cataract surgery performed by the same surgeon. All patients were informed about the operation, and each provided a written informed consent to participate in the study.

To be included in the sample, patients needed to be 40 to 90 years old and have undergone cataract surgery. Exclusion criteria were previous ocular or intraocular surgery, evidence of trauma, acute or chronic corneal infection, inflammation of the cornea during slit lamp examination and intraoperative or postoperative complications. Patients with a history of any other ocular disease that could affect visual outcomes (e.g., color vision impairment or chronic uveitis) or contrast sensitivity (e.g., glaucoma, maculopathy, or high myopia) were also excluded.

Surgical technique

All operations were performed under local anaesthesia. After anaesthesia, a transparent corneal incision was made using a 2.2 mm blade, and sodium hyaluronate was given to the anterior chamber as the viscoelastic material and iris retractor was inserted to the patients who were not dilated sufficiently. Capsulorhexis was performed after sufficient hydrodissection, cataract emulsification was performed using the stop and chop technique, which produced the least strain on the capsules and zonules. Following bimanual irrigation-aspiration, monofocal acrylic IOL was implanted with viscoelastic material. CTR was placed with the help of manual forceps at any stage of the surgery where zonular weakness was noticed.

For eyes in Group 1, white-to-white (WTW) corneal diameter was considered in selecting CTRs (OPHTEC, Groningen, the Netherlands), all of which consisted of a single-piece polymethyl methacrylate ring with open rings at each end. Two models of CTRs were used: Model 275, with uncompressed and compressed diameters of 12 and 10 mm, respectively, and Model 276, with uncompressed and compressed diameters of 13 and 11 mm, also respectively. Model 275 was implanted in eyes with WTW diameters up to 11.5 mm, whereas Model 276 was implanted in eyes with WTW diameters exceeding 11.5 mm. In each eye in Group 2, the IOL was injected into the capsular bag without the insertion of a CTR.

Data Collection

The IOL calculation of the cases was performed by optical biometry instrument (AL_Scan, Nidek Co. Ltd. Gamagori, Japan) using the SRK-T formula. IOL calculation was made with the aim of emmetropia. Preoperative keratometry values, WTW measurements, anterior chamber depths (ACD), axial lengths (AL) and refraction measurements were made in all cases.

Refraction measurements were made 1 month after the operation. The refractive prediction error (RPE) was used as a measure of refractive outcome accuracy and was obtained by subtracting the predicted refractive error from the postoperative manifest refraction. Therefore, a positive value indicated a hyperopic shift from the predicted refraction. A negative value was evaluated as myopia shift. The results were evaluated according to 3 criteria: mean ArRPE; mean AbRPE; ranges and number of eyes within a certain RPE (± 0.50 D, ± 1.00 D and ± 2.00 D). 

Statistical Analysis

Statistical analysis was conducted using PASW Statistics (version 15, SPSS Inc.), with the Kolmogorov–Smirnov test used to confirm the normal distribution of the data. To compare ages, AL, ACD, WTW diameter, IOL power and keratometry values between the groups, an independent t test was performed. By contrast, the chi-square test was used to compare the sex and laterality of the groups. Results were statistically significant when their p values were less than 0.05.

## Results

Each 29 eyes from 29 patients, Group 1 (i.e., patients with CTRs) and Group 2 (i.e., patients without CTRs) were sex-matched such that each group contained 13 women and 16 men. Whereas the mean age in Group 1 was 72.2 years (range: 38-90 years), the mean age in Group 2 was 70.6 years (range: 54 to 85 years). In Group 1, the aetiology of zonular instability was most often idiopathic.

All 58 patients underwent phacoemulsification using a similar technique performed by the same surgeon. Decisions to insert CTRs or not were made intraoperatively, and successful IOL and/ or CTR implantation was possible in all cases.

**Table 1** shows the preoperative patients’ demographic data. There was no statistically significant difference in the mean age, corneal power, AL, and ACD (**Table 1**).

**Tabel 1:** Patient demographic data and preoperative ocular dimension

GROUP	CTR	CONTROL	P value
Number of subject	29	29	
Number of eyes	29	29	
Age (yrs)	72.72 (38-909)	70.66 (54-85)	*p*=0,438
Female, n (%)	13	13	*p*=1,0
Left eye, n (%)	11	14	
Mean K (D)	43.78 ( 40.32–47.67)	44.15 (40.37–46.94)	*p*=0,382
Mean AL (mm)	23.57 (22.14–26.26)	23.49 (21.90–25.83)	*p*=0,738
Mean HWTW (mm)	11.84 (11.0–12.4)	11.44 (8.1–12.9)	
Mean ACD (mm)	3.07 (1.92–5.21)	3.14 (2.47–3.83)	*p*=0,580
CTR = Capsular tension ring, K = Keratometry, AL = Axial lengths, WTW = White-to-white, ACD = Anterior chamber depths			

**Table 2** presents the mean arithmetic refractive prediction errors (ArRPE) and mean absolute refractive prediction error (AbRPE) in Group 1 versus Group 2. No statistically significant difference between Group 1 and Group 2 emerged in ArRPE (0.52 vs. 0.45 D, p = 0 .570) or AbRPE (0.52 vs. 0.55 D, p = 0.799). Calculations with the SRK/ T formula also revealed no statistically significant between-group difference in mean ArRPE or AbRPE (**Table 2**). Postoperative hypermetropic shift occurred in most cases in both groups, although the mean difference between eyes in Group 1 (22/ 29) and Group 2 (25/ 29) was not statistically significant (p = 0.315).

**Tabel 2:** Mean arithmetic and absolute refractive prediction error using SRK/ T comparing CTR and control groups

	CTR	CONTROL	
Arithmetic RPE			
Mean	0.52 ± 0,41	0,45 ± 0,52	P=0.570
Median	0.40	0,44	
Range	0,35-1,750	-0,85-1,710	
Variance	0,167	0,269	
Absolute RPE			
Mean	0.52 ± 0,41	0,55 ± 0,41	P=0.799
Median	0.40	0,47	
Range	0,35-1,750	0,10-1,710	
Variance	0,167	0,167	
	CTR	CONTROL	
Arithmetic RPE			
Mean	0.52 ± 0,41	0,45 ± 0,52	P=0.570
Median	0.40	0,44	
Range	0,35-1,750	-0,85-1,710	
Variance	0,167	0,269	
Absolute RPE			
Mean	0.52 ± 0,41	0,55 ± 0,41	P=0.799
Median	0.40	0,47	
Range	0,35-1,750	0,10-1,710	
Variance	0,167	0,167	
CTR = Capsular tension ring, RPE = Refractive prediction errors			

**Table 3** shows the percentage and number of eyes within ± 0.5 D, ± 1.0 D, and ± 2.0 D of the predicted refraction with the SRK/ T formulas in the CTR group and the control group. The percentage of eyes in the ± 1.0 D range of the predicted refraction in both groups was 90%. Three patients in both groups had a prediction error greater than 1.0 D.

**Tabel 3:** Number of eyes within a certain refractive prediction error using SRK/ T

	Within ± 0.5 D	Within ± 1D	Within ± 2D	Myopic shift	Hypermetropic shift
CTR	17 (%59)	9 (%31)	3 (%10)	7 (24)	22 (76)
CONTROL	15 (%52)	11 (%38)	3 (%10)	4 (14)	25 (86)
TOTAL	32	20	6	11	49
CTR = Capsular tension ring					

## Discussion

Although developed to stabilize the capsular bag, CTRs are implanted during cataract surgery to not only stabilize the capsular bag’s position but also ease phacoemulsification and center the IOL after surgery in eyes with zonular dehiscence and compromised capsular bag stability amid zonular weakness. CTRs can also be implanted to reduce the risk of vitreous prolapse, capsular rupture and the postoperative dislocation of the IOL.

This study was performed to clarify whether the implantation of a CTR during phacoemulsification in zonular instability affects the refractive outcomes. In our study, the implantation of a CTR did not induce a significantly higher hyperopic or myopic shift compared with results in a control group in which no CTR was implanted. According to the results of our study, there was no need to modify the IOL calculations when CTR implantation was planned. 

Sun and Gimbel have developed an in vitro study, which controls the position of CTR and indicates that CTR is unlikely to affect the IOL power formula as it does not change the position of the IOL [**15**]. Findl et al. measured the effective lens position using dual-beam PCI and found the lens-capsule distance was almost identical in eyes with a silicone IOL with or without implantation of a CTR [**16**].

Boomer and Jackson did not find a statistically significant difference in refraction errors in a retrospective case-control study in which 19 eyes underwent CTR compared with 24 eyes without zonular instability. They reported that there was no consistent effect of CTR on refractive outcome, and it was unnecessary to modify the IOL calculations [**17**]. In this study, Boomer and Jackson had refractive results in ± 1.0 D in all eyes with CTR and 96% (Holladay 2) and 88% (SRK/ T) in the control group. Similarly, in our study, 90% of the eyes with CTR and control group had refractive outcome in 1.0 D. In our study, 58% of the eyes with CTR and 52% of the control group had a refractive outcome in ± 0.5 D.

Postoperative changes in refractive outcomes are known to relate to IOL decentration and tilt. In response, CTR implantation can avert the contracture of the capsular bag and thus prevent IOL decentration [**4**]. Among authors who have sought to determine the effect of CTRs on refraction outcomes, Schild et al. demonstrated that, following CTR placement, the CTR and the IOL remained in the capsular bag when the CTR was positioned between the IOL haptics and the ciliary body [**18**]. In our study, comparing refractive outcomes in myopic eyes with and without CTRs following phacoemulsification, no statistically significant difference arose in AbRPE between Groups 1 and 2 [**19**]. Our results also showed, as did Boomer and Jackson’s, that IOL power can be calculated as usual even when CTRs are used.

In our study, no statistically significant difference surfaced between the groups in rates of postoperative hyperopic and myopic shifts. However, statistically significant hypermetropic shift was observed in both groups. At the same time, though Group 1 contained a greater number of such shifts, the between-group difference was not statistically significant, nor was the between-group difference in AbRPE. Thus, our results support the findings of the four studies cited above.

In another work, examining 52 patients who had undergone cataract surgery, Park et al. compared 26 eyes with CTRs and 26 without the rings. Co-implanting CTRs and IOLs induced more hyperopic refractive outcomes than implanting IOLs alone. However, because the effect of CTRs may be inconsistent across different IOL designs and materials, the calculated IOL power should be reviewed [**20**].

From another angle, Baranwal et al. performed ultrabiomicroscopy to determine the shift in IOL in eyes with CTRs versus the ones without them [**21**]. Among their results, ultrabiomicroscopy revealed a posterior shift of IOLs after CTRs were used, which later required postoperative hypermetropic correction. Thus, they suggested that the posterior shift of IOLs following the use of CTRs should be considered and that IOLs should be +1.0 to 2.0 D more than what was calculated preoperatively.

The limitations of our study were its retrospective approach and lack of ultrabiomicroscopy to measure effective IOL-capsule distance and effective IOL position. No difference in refraction was observed in eyes that received CTRs, which might have caused the change in IOL.

## Conclusion

Our results suggest that CTRs are not only tools to stabilize the zonular apparatus and capsular bag during complicated cataract surgeries but also devices able to deliver more accurate refractive outcomes in eyes. If improving fracture outcomes is a goal of the cataract surgeon, then CTRs should be considered, even in cases of mild zonular instability. Despite arguments that CTRs should not be placed because improper placement can further deteriorate capsules and zonules, in the hands of a skilled, experienced surgeon, CTRs can be safely implanted with relative ease.

**Conflict of Interest**

The authors declare no conflict of interest.

**Informed Consent and Human and Animal Rights statements**

Informed consent has been obtained from all individuals included in this study.

**Authorization for the use of human subjects**

Ethical approval: The research related to human use complies with all the relevant national regulations, institutional policies, is in accordance with the tenets of the Helsinki Declaration, and has been approved by the Ethics Committee of Kutahya Health Sciences University School of Medicine, Kutahya, Turkey.

**Acknowledgements**

None.

**Sources of Funding**

None.

**Disclosures**

None. 
